# Pediatric Tuberculosis: A Review of Evidence-Based Best Practices for Clinicians and Health Care Providers

**DOI:** 10.3390/pathogens13060467

**Published:** 2024-06-01

**Authors:** Brittany K. Moore, Stephen M. Graham, Subhadra Nandakumar, Joshua Doyle, Susan A. Maloney

**Affiliations:** 1 Division of Global HIV and Tuberculosis, U.S. Centers for Disease Control and Prevention, Atlanta, GA 30329, USA; ifd0@cdc.gov (S.N.); xzd2@cdc.gov (J.D.); smaloney@cdc.gov (S.A.M.); 2 Centre for International Child Health, Department of Pediatrics, University of Melbourne, Melbourne 3052, Australia; steve.graham@rch.org.au; 3 Murdoch Children’s Research Institute, Royal Children’s Hospital, Melbourne 3052, Australia; 4 International Union Against Tuberculosis and Lung Disease, 75001 Paris, France

**Keywords:** pediatric, tuberculosis, infectious disease

## Abstract

Advances in pediatric TB care are promising, the result of decades of advocacy, operational and clinical trials research, and political will by national and local TB programs in high-burden countries. However, implementation challenges remain in linking policy to practice and scaling up innovations for prevention, diagnosis, and treatment of TB in children, especially in resource-limited settings. There is both need and opportunity to strengthen clinician confidence in making a TB diagnosis and managing the various manifestations of TB in children, which can facilitate the translation of evidence to action and expand access to new tools and strategies to address TB in this population. This review aims to summarize existing guidance and best practices for clinicians and health care providers in low-resource, TB-endemic settings and identify resources with more detailed and actionable information for decision-making along the clinical cascade to prevent, find, and cure TB in children.

## 1. Introduction

One quarter of the world’s population—1.7 billion people—is infected with tuberculosis (TB), including 67 million children (defined throughout as persons under 15 years of age) [1,2,3]. In 2022, 1.25 million children developed TB, and 214,000 children died from this preventable, curable disease, representing 12% of the global burden of TB disease and 17% of all deaths from TB [4]. An estimated 80% of these deaths are in children under five years of age, and 96% are in children who never received TB treatment [5]. Children, especially those under five years of age and those with HIV infection, severe acute malnutrition (SAM), or other immunocompromising conditions, are more likely to progress rapidly from infection to severe disease and are more likely than adults to die from TB. Such rapid progression provides a narrow window for intervention to prevent disease and death [5,6,7,8,9,10,11,12].

The disproportionate TB burden in this age group is due to unique vulnerabilities, varying clinical presentations, and challenges in screening for, diagnosing, treating, and preventing TB in children. A schematic of the pathway through TB exposure, infection, and disease in children and potential areas of challenge where children are lost along the clinical care cascade is included in the World Health Organization’s (WHO’s) Roadmap to ending TB in children and adolescents [13]. For instance, children with TB often present with non-specific clinical signs and symptoms resembling common childhood illnesses, seek care at health clinics or departments that do not routinely screen to identify presumptive TB, and/or do not have access to recommended diagnostic tests and radiography services to evaluate those identified with presumptive TB. Additionally, young children are more likely to have extrapulmonary TB (EPTB), which further increases variability in their clinical presentation. These challenges may hinder the appropriate evaluation of children for TB disease [6,14,15,16]. Children have difficulty providing high-quality, spontaneously expectorated sputum for diagnostic testing, especially young children, and often require more invasive sampling procedures such as sputum induction or gastric aspiration [6,14,17,18]. Consequently, increased use of less invasive specimens (e.g., stool, urine, nasopharyngeal aspirates (NPA)) is now recommended. However, these specimen types often have fewer mycobacteria, resulting in decreased test sensitivities for young children [19,20]. Clinical diagnosis based on signs, symptoms, patient history, and radiography therefore remains critically important for identifying TB in children [18,21]. Furthermore, clinicians often need to use every piece of information available to guide a clinical decision on TB treatment for pediatric patients with presumed TB but with negative diagnostic test results (see Treatment Decision Algorithms below).

Once initiated on treatment, children respond well to preventive and curative treatment regimens for drug-susceptible (DS) and drug-resistant (DR) TB, generally experiencing fewer and less severe side effects and better treatment outcomes than adults if treatment completion can be assured [6,18,22]. This better response to treatment underscores the opportunities lost due to challenges in TB case-finding for children. Fortunately, a greater focus on TB in children and adolescents, increasing confidence and familiarity with clinical diagnosis of TB in children, and recent advances in TB diagnostic testing, including the use of child-friendly specimens, have contributed to a four-fold increase in pediatric TB case detection during the last decade [4,13]. Moreover, there has been a growing recognition of the need to prioritize and strengthen pediatric TB prevention and treatment programs to decrease morbidity and mortality in children. Routine, systematic TB screening in settings where children seek care and child-focused case-finding efforts, like household contact investigations, can prevent disease and death by facilitating the provision of TB preventive treatment (TPT) or early diagnosis and appropriate treatment of TB disease [14,16,20,23,24,25,26,27]. There have also been advances in the development and accessibility of pediatric formulations for both TB preventive and disease treatment regimens, which have been procured by many programs in high-burden countries [28,29,30]. Finally, WHO recently released updated global guidelines on the management of TB in children and adolescents, recommending several shorter treatment regimens for children and young adolescents with non-severe drug-susceptible TB, TB meningitis (TBM), and DR TB [31,32,33].

But challenges remain, and in 2022 globally, only 42% of children under five years of age and 55% of children ages 5–14 years with TB disease were diagnosed, compared with 70% of adults [4]. Among the estimated 25,000 to 32,000 children who develop DR TB every year, only 14% were diagnosed [4]. These challenges have only grown in the wake of the COVID-19 pandemic, which led to limited access to TB services, fewer new TB diagnoses (which were more pronounced among children), a decrease in bacille Calmette–Guérin (BCG) vaccine availability and coverage, and an overall increase in mortality [1,34,35,36]. Global TB cases and deaths now resemble the burden of disease in 2016, erasing enormous progress realized in the years leading up to the COVID-19 pandemic.

Advances in pediatric TB care are promising overall, the result of decades of advocacy, operational and clinical trial research, and political will among national and local TB programs in high-burden countries. However, implementation challenges remain in translating policy to practice and scaling up innovations for prevention, diagnosis, and treatment of TB in children, especially in low-resource settings. There is also both need and opportunity to strengthen clinician confidence in making a TB diagnosis and managing the various manifestations of TB in children, which can facilitate the translation of evidence to action and expand access to new tools and strategies to address TB in this population. This review aims to summarize existing guidance and best practices for clinicians and health care providers in low-resource, TB-endemic settings and identify resources (Table 1) with more detailed and actionable information for decision-making along the clinical cascade to prevent, find, and cure TB in children.

## 2. Prevention of TB in Children

The risk of developing disease after TB infection (TBI) in children is associated with numerous factors, including age at exposure, immune and nutritional status, genetics, and pathogen virulence. A recent modeling study estimated that HIV infection, undernutrition, and a lack of bacille Calmette–Guérin (BCG) vaccination in TB-endemic settings may account for 25% of new TB cases in children [37]. Young age and HIV infection are the most important risk factors for severe or disseminated disease, such as TBM and miliary TB, which are the most common causes of death from TB in children. Children who develop TB disease usually do so within one year following infection; therefore, TB in children is also an indicator of ongoing transmission within a household or the community. The risk of progression from untreated TBI to TB disease varies by age [38]. In a recent meta-analysis of children with close TB exposure, the two-year risk of developing TB in children with positive TBI tests who had not received TPT was 19% for children under five years of age, 9% for children 5–14 years of age, and 11% in adolescents 15–18 years of age [9]. The risk of developing TB was highest during the first 90 days after exposure or evaluation and in the youngest children; 83% of children under five who developed TB did so within 90 days of their initial visit, underlining the importance of prompt contact investigations and initiation of TPT [9]. 

The main interventions currently available to reduce the risk of TBI and/or progression to active TB disease in children are: (1) community-based active case detection and treatment of people with TB (and TBI), (2) vaccination with BCG, (3) TPT, and (4) TB infection prevention and control [14,39]. There are also numerous new TB candidate vaccines in development—some of which are in advanced clinical trials—offering the prospect of new interventions on the horizon, which will be discussed later in the manuscript.

### 2.1. Community-Based Active TB Case Detection and Treatment for TB and TBI

One important population-based intervention that can reduce the risk of TB exposure and infection (and thereby TB disease) in children is a reduction in the overall number of TB cases in a population through community-wide screening, active case finding, and treatment for TB disease and TBI. The successful impact of these population-based interventions in reducing TB transmission and prevalence was demonstrated in Alaska, USA, more than 50 years ago [40]. A recent cluster-randomized controlled study (called ACT3) evaluated the effectiveness of active, community-wide TB disease screening and treatment for adults in reducing TB and TBI prevalence in a high-TB burden province in Vietnam [41]. At the end of the ACT3 study period, researchers found a statistically significant reduction (44%) in TB prevalence in the intervention clusters receiving community-based active screening and treatment for TB disease when compared to standard passive TB case detection alone. Furthermore, although this study did not find a statistical difference in TBI among young children born immediately before the interventions began, post ad-hoc analyses among older children (3–10 years of age at the start of the intervention) demonstrated a 50% reduction in the prevalence of TBI in these children when compared to children in control clusters. This study suggests that older children, who perhaps had more interactions in the community, benefited most from decreased TB transmission [41]. Other modeling studies and randomized clinical trials, either underway or completed, have taken a holistic case-finding approach, finding and treating both active TB disease and TBI, to rapidly reduce TB in a durable way [42,43].

### 2.2. BCG Vaccine

BCG vaccine, the only vaccine currently licensed for the prevention of TB, is a live, attenuated vaccine using Mycobacterium bovis (a mycobacterium species that causes TB in cattle) launched over 100 years ago. In 2020, WHO reported that BCG vaccine was part of the national childhood vaccine schedule in 154 countries, with most countries reporting coverage of at least 90% [44]. With millions of doses administered to infants globally every year, BCG is one of the most widely used vaccines in the world and is considered highly cost-effective in low-resource countries with high-TB burdens [45,46,47,48]. There have been numerous efficacy trials and epidemiological studies of BCG over the past several decades [47,48,49,50,51,52,53,54,55,56]. The greatest benefit of BCG vaccination, which drives its cost-effectiveness [48], is its efficacy in prevention of severe forms of TB (e.g., TBM and miliary TB), especially in young children. Randomized clinical trials (RCTs) indicate that BCG has 85% protective efficacy against severe forms of TB, with the highest protection found in infants immunized during the neonatal period (90% reduction in severe forms of TB) [47].

Worldwide, several BCG vaccines, based on different BCG strains, are available and administered by intradermal injection. BCG vaccination typically causes a scar at the site of injection; however, scar formation is not a marker for protection, and there may be differences in protective efficacy between vaccine strains. BCG vaccine can cause more severe, localized reactions, including hypersensitivity, ulceration, abscess formation, and regional lymphadenitis, but this is uncommon. Most of these adverse events are self-limited and do not require medical or surgical intervention. Disseminated BCG disease, which is associated with an overall case-fatality rate of up to 70% and was rare in the pre-HIV era (one case per 1 million BCG-vaccinated children), currently occurs mainly in children living with HIV (CLHIV) and in those with primary immunodeficiencies. Rates of disseminated BCG in children increased following the advent of the HIV epidemic and prior to early antiretroviral therapy (ART), with one South African study reporting a rate of 992 per 100,000 BCG-vaccinated CLHIV [57,58]. Fortunately, these rates have fallen following implementation of early ART for HIV-infected infants and are again a rare phenomenon. Disseminated BCG disease presents a clinical picture similar to TB, with findings including failure to thrive, anemia, hepatosplenomegaly, lymphadenitis, osteomyelitis, and pneumonia. BCG-derived M. bovis yields a positive result on initial molecular tests, such as Xpert MTB/RIF (Xpert) and Xpert MTB/RIF Ultra (Xpert Ultra), so culture may be necessary to distinguish it from TB [59]. Management of disseminated BCG disease usually includes treatment with isoniazid, rifampicin, and ethambutol (with or without a fluoroquinolone such as levofloxacin), as BCG is resistant to pyrazinamide and some strains are partially resistant to isoniazid [39,60].

WHO currently recommends that a single dose of BCG vaccine be given to all healthy neonates at birth (or at the earliest opportunity thereafter) for prevention of TB in countries and settings with a high incidence of TB, defined by WHO as more than 100 cases of TB per 100,000 population [61]. Countries with a low incidence of TB may choose to selectively vaccinate high-risk neonates or other high-risk populations, such as immigrants from high-TB-incidence countries and health care workers, which has been found to be more cost-effective than universal BCG vaccination in these settings [50,59,62,63,64,65]. While CLHIV, when vaccinated with BCG at birth, are at increased risk of developing disseminated BCG disease [57], after a risk-benefit analysis, WHO guidelines now recommend that neonates and CLHIV in high TB incidence settings receive BCG vaccination if they are stable on ART (CD4% > 25% for children aged <5 years or CD4 count ≥ 200 cells/mm^3^ if aged >5 years) or clinically stable if CD4 testing is unavailable. The timing of BCG vaccine administration is important to consider in infants exposed to HIV, infants living with HIV, and neonates exposed to mothers with infectious TB [14,39,47]. 

While BCG vaccine is known to be effective in protecting infants and children from severe TB disease, demonstrated protection has not been consistent against all forms of TB and in all age groups. Protection also varies by geographic latitude (which may be a marker for atypical mycobacterial prevalence). BCG vaccine efficacy against pulmonary TB (PTB) has varied widely across studies, with lower efficacy in adults, although some recent evaluations have found higher efficacy in certain groups [51]. Evidence also indicates that BCG vaccine protects against TBI as well as TB disease; it has been estimated that BCG vaccine may prevent approximately 20% of vaccinated children from developing TBI following exposure to TB [47,52]. However, there is no evidence of effectiveness when BCG vaccine is used as a post-exposure prophylaxis after TBI [51,54,56]. BCG vaccine also demonstrates some effectiveness against leprosy, Buruli ulcer, and non-tuberculous mycobacteria (NTM) lymphadenitis in children as well as non-specific (likely immunologically mediated) effects associated with decreased all-cause mortality rates, especially in children [39,47,66]. Protection after primary BCG vaccination could last for up to 15 years; however, overall vaccine efficacy declines over time [47,52]. 

### 2.3. TB Preventive Treatment 

TBI is defined by WHO as “a state of persistent immune response to stimulation by Mycobacterium tuberculosis antigens with no evidence of clinically manifested active TB disease” [61]. An estimated 7.5 million children are infected with TB each year [67,68]. There are more than 67 million children infected with TB worldwide, including 2 million children infected with multidrug-resistant TB (MDR-TB) and 100,000 children with extensively drug-resistant TB (XDR-TB) [1]. After being exposed to a person with TB disease at home, it is estimated that up to 35% of children under five years of age will develop TBI and up to 10% will develop TB disease [27]. Such findings underscore the importance of contact investigations in identifying and screening children who have been exposed to TB, which can greatly enhance TB and TBI case-finding, evaluation, and treatment [27,69]. In addition, CLHIV are 8–20 times more likely to develop TB disease than those without HIV infection, and this risk remains elevated, although blunted, in CLHIV on ART [14]. Providing treatment for TBI to prevent progression to TB disease (TPT) is an effective and critical component of efforts to end TB locally and globally [70].

Despite the importance of TPT, from 2018 to 2022, only 15.5 million people were treated with TPT globally. Most (11.3 million) were people living with HIV (PLHIV) treated through the U.S. President’s Emergency Plan for AIDS Relief (PEPFAR)-supported programs [4], including nearly 600,000 CLHIV [71]. However, far fewer eligible household contacts receive TPT; in 2022, only 37% of eligible household contacts under five years of age and only 11% of contacts above five years of age were provided TPT [4]. Most children who would benefit do not receive TPT either because they are not appropriately identified or because TPT is out of stock. Implementing an effective TB prevention program requires a comprehensive package of interventions: (1) identification, screening, and testing in populations with high TB risk; and (2) delivering and monitoring effective and safe preventive treatment. WHO has developed an application for monitoring this cascade of care called the PREVENT-TB tool [39], which is also included in the Resources Table (Table 1).

#### 2.3.1. Identification, Screening, and Evaluation of Children at High Risk of TB

**Figure 1:**
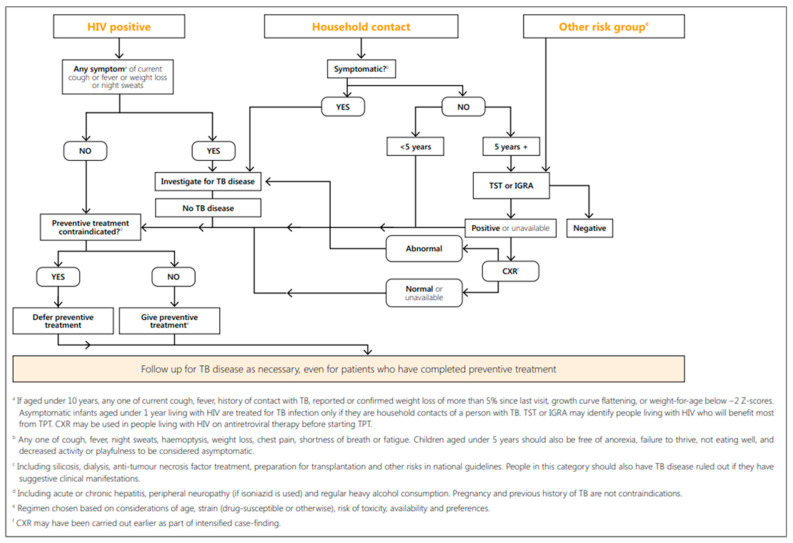
The WHO algorithm for screening for and evaluation of TBI and TPT eligibility in children from the WHO operational handbook on tuberculosis. Module 5: management of tuberculosis in children and adolescents [39].

WHO recommends TB contact investigations as well as systematic screening for children at high risk for TB disease and TBI in all clinical settings where children seek care [14]. Systematic screening includes identifying people at risk for TB disease in a predetermined target group by assessing signs and symptoms and using tests, examinations, or other procedures that can provide rapid results to determine if further evaluation is needed. In children who screen positive, one or more diagnostic tests and clinical assessments are usually required to establish a TB diagnosis (see Section 3). Screening can also identify children who are eligible for and could benefit from TPT if the child does not have TB disease [14,39]. For all children, it is important to determine that a child does not have TB disease before initiating TPT. Screening approaches differ slightly for the two primary groups of children at high risk of TB: (1) those in close contact with a person with TB, and (2) those with elevated risk of progression from TBI to TB disease, including young children, CLHIV, children with SAM, and other risk groups [14,39,72]. The WHO-recommended TBI and TPT eligibility algorithm (Figure 1) outlines the steps involved in making a decision to initiate TPT [39].

WHO currently recommends that child and adolescent household contacts of people with bacteriologically confirmed TB disease should be systematically screened for TB disease during contact investigations [61]. Recommended screening includes symptom screening (with symptoms including cough for more than two weeks, fever for more than two weeks, poor weight gain [or weight loss] in the past three months, or reduced playfulness or lethargy in young children) and/or a chest X-ray (CXR) (both posteroanterior and lateral views). For those children under five years of age who are screened and found not to have TB disease, WHO recommends a course of TPT. For older children and adolescents who have been found not to have TB disease, WHO recommends consideration for TPT as well, if resources allow.

WHO also recommends that CLHIV be systemically screened for TB disease at each visit to a health facility and evaluated for TPT in all settings [14,39]. For CLHIV, screening (with symptoms including current cough, fever of any duration, and poor weight gain in the past three months) is recommended during every encounter with a health care worker. There are additional situations in which CLHIV are prioritized for immediate evaluation, regardless of symptoms.

Triggers for Diagnostic Evaluation for CLHIV (See also Section 3) [14]:

- If CLHIV screen positive on a TB symptom screen, they should be immediately flagged for further diagnostic evaluation for TB disease.
- If CLHIV have a documented contact with someone with TB disease, they are prioritized for immediate diagnostic evaluation, regardless of symptoms.
- For CLHIV with advanced HIV disease (AHD) or who are seriously ill, lateral flow urine lipoarabinomannan assays (LF-LAM) can be used as an initial TB test, regardless of the presence of symptoms.

Provision of TPT when the TB screen is negative in CLHIV [14]:

- CLHIV over 12 months of age who have a negative TB screening evaluation and live in a high TB burden setting should be offered TPT as part of a comprehensive package of HIV care, regardless of history of contact with a person with TB.
- For infants under 12 months of age living with HIV, TPT is recommended if there is a history of close contact with a person with TB and the infant has had a negative screen for TB disease.

It should be noted that tuberculin skin tests (TST) and interferon-gamma release assays (IGRA) have only limited utility as screening tools for TB disease in children (or adults), as both tests are unable to distinguish between TBI and TB disease. Both tests can serve as markers for TBI; however, they can each give false-positive and false-negative results [73,74]. TBI testing by TST or IGRA is not required before initiating TPT for household contacts under five years of age or CLHIV. These tests play a role in decision-making for TPT initiation (Figure 1) and in an extended diagnostic evaluation of children with presumed TB(discussed further in the Section 3).

#### 2.3.2. TPT Guidance and Implementation Considerations for Children

TPT is a mainstay of TB prevention in children, as TPT can prevent TB disease in 91% of children and adolescents with TBI [38,39]. Further, a recent study modeled the combined intervention of contact investigations and TPT in 29 high-incidence countries and found it was cost-effective for household contacts of all ages, saving an estimated 850,000 lives (700,000 among household contacts under 15 years) by 2035 if short-course TPT was provided to all contacts who screened negative for TB [75]. 

There are several short and effective TPT regimens recommended by WHO, following clinical trials that included children and adolescents (Table 2) [38,39,76]. WHO currently recommends the following TPT options for use in children, although there are special considerations for CLHIV (see below);

- Six or nine months of isoniazid daily (6H or 9H) (all ages);
- Three months of weekly isoniazid plus rifapentine (3HP);
- Three months of isoniazid plus rifampicin daily (3HR) (all ages);
- One month of daily isoniazid plus rifapentine (1HP); or;
- Four months of daily rifampicin (4R) (all ages) [39].

The age of the child, HIV status, ART regimen, and availability and affordability of child-friendly formulations have bearing on the choice of TPT regimen. Compared to 6H, the newer rifamycin-based regimens demonstrate equal efficacy with better adherence, less drug-related toxicity, and potentially lower costs due to decreased utilization of the health system. Rifampicin- and rifapentine-containing regimens should be prescribed with caution in CLHIV on ART because of potential drug–drug interactions with the most common ART regimens. Although pharmacokinetic and safety studies are underway to inform use of these regimens in this population, 6H is currently the preferred regimen for CLHIV under 13 years [14,39]. WHO guidelines are expected in July 2024 regarding use of rifapentine and dolutegravir [77]. Globally, 3HR is the preferred TPT option among HIV-negative children because child-friendly dispersible fixed-dose combinations (FDCs) for rifampicin (75 mg) and isoniazid (50 mg) are widely available, used for TB treatment, and are less prone to dosing errors than single formulations. Pediatric formulations are unavailable in some countries, especially those with low TB incidence, so crushing of pills, opening capsules, or compounding are utilized to ensure correct dosing [38]. Monitoring and evaluating children on TPT can take place at a health care facility, in the community (by treatment supporters), or by using digital tools such as video-supported treatment [23,39,78] at recommended monitoring intervals noted in Table 2.

In high-risk household contacts of people with MDR-TB, WHO notes that TPT “may be considered based on individualized risk assessment and sound clinical justification” [14]. WHO is currently updating guidelines regarding specific TPT regimens for children who are contacts of persons with MDR-TB; they are expected by July 2024. In the interim, a recent Rapid Communication from WHO stated that a regimen of six months of levofloxacin should now be used as TPT for contacts of MDR or rifampicin-resistant (RR) TB patients based on evidence from two well-conducted randomized clinical trials in South Africa and Vietnam [14,39,61,77].

### 2.4. TB Infection Prevention and Control

Infection prevention and control (IPC) in health care services and other settings at high risk for transmission is a pillar of global TB prevention efforts. Children with TB are often considered unlikely to transmit TB (due to paucibacillary disease and a relative lack of cough) [6,79], but children can and do transmit TB [6]. Further, both children and adults are at risk for TB transmission in health care facilities and households without adequate infection control measures in place. Therefore, the same IPC principles apply for both children and adults in health care facilities and other high-risk transmission settings, including in areas dedicated solely to children. IPC recommendations to reduce TB transmission in these settings include:

1. administrative controls (e.g., fast-tracking people with TB symptoms for evaluation, isolating those with presumptive or diagnosed TB disease, initiating TB treatment immediately for those with TB, and encouraging cough etiquette and providing masks for anyone with an active cough or presumed TB);
2. environmental controls (e.g., natural and mechanical ventilation systems, and upper-room germicidal ultraviolet systems); and
3. respiratory protection for health care workers (HCWs) (e.g., N95 masks/particulate respirators) [80].

The greatest risk of transmission to children in health facilities occurs when young children mix with adults and adolescents with untreated TB. Therefore, people with cough and/or presumptive TB should not mix with infants and children in child health settings (e.g., immunization clinics or well-baby checks) or children and adolescents at HIV clinics. In high-burden settings, there is a particularly high risk that adults accompanying or visiting children may have untreated TB disease [39].

## 3. Evaluating Children with Presumptive TB

Diagnostic evaluation is the next step in the care cascade after a child has screened positive for TB. In practice, diagnosing TB in children necessitates using all available findings from clinical assessments, radiography, and laboratory investigations to inform a treatment decision. Since current diagnostic tests have lower sensitivity in children, especially young children and CLHIV (see Table 3), most of these children are diagnosed based on a careful medical history and clinical examination, along with CXR and TBI testing when available. Bacteriologic confirmation of TB disease in these children is important and utilizing the highest number and combination of traditional and alternative laboratory specimen types, including those that are more child-friendly and less invasive, can improve the likelihood of obtaining confirmation [81]. Such efforts are important to guard against overdiagnosis and ensure pediatric patients have access to drug resistance testing. Older adolescents who present with TB symptoms similar to those in adults and can produce sputum and other specimen types recommended for adult patients can be evaluated using approaches designed for adults [73].

In general, evaluating a child for TB relies on a combination of the following:

- Clinical assessment,
- Careful medical history, including contact with someone who had TB, previous TB treatment, BCG vaccination, and signs and symptoms consistent with TB;
- Clinical examination, including growth assessment; and
- HIV testing if the status is unknown.
- TBI testing (TST or IGRA),
- Radiological investigations, and
- Bacteriological investigations, including those relevant to presumed EPTB.

We describe below WHO-recommended clinical assessment approaches as well as radiologic and bacteriological investigations and their applications. Also highlighted and discussed is the importance of integrated clinical decision algorithms designed to standardize and score the findings from these investigations to inform a treatment decision.

### 3.1. Clinical Assessment

When evaluating a child for TB, it is important to perform a careful medical history, which includes information about history of contact with someone who has or had TB, visitation or residence in a high TB incidence community, previous TB treatment, BCG vaccination, and signs and symptoms consistent with TB. It is also important to consider nutritional status and risk factors for TBI and disease, including young age, HIV infection, SAM, recent history of measles, and the presence of other immunocompromising conditions or immunosuppressive therapy [39]. If a TB source case is identified, treatment regimen and treatment response for the source patient should be sought. Any child or adolescent with presumed or confirmed TB should be tested for HIV if their status is unknown.

Although there are no clinical examinations that can definitively confirm TB, a variety of both nonspecific and specific signs should raise clinical suspicion, prompt a thorough evaluation for TB disease, and also inform clinical decision-making on TB treatment [39,93].

Children most commonly present with PTB. In younger children, PTB usually involves disease in the intrathoracic lymph nodes, while PTB more commonly resembles adult-type disease in older children and adolescents, with parenchymal lung involvement, and it may include cavitary lesions in severe cases [14,39]. Common symptoms of PTB in children include persistent cough, weight loss and/or poor weight gain, prolonged fever and/or night sweats, fatigue, reduced playfulness, and lower activity levels. There should be a high index of suspicion for TB if symptoms are persistent (longer than two weeks), especially following treatment for differential diagnoses (e.g., antibiotics for pneumonia, antimalarials for fever, nutritional rehabilitation for failure to thrive or malnutrition) [14,39]. In children with PTB, auscultation and percussion of the lungs can be normal but may reveal lung disease (e.g., crackles, bronchial breathing, or wheezing). Other clinical findings suggestive of PTB include acute pneumonia (especially in CLHIV and severely malnourished children), persistent wheeze not responding to bronchodilators, and SAM, especially if not responding to therapeutic nutritional treatment [14,39,93].

**Figure 2:**
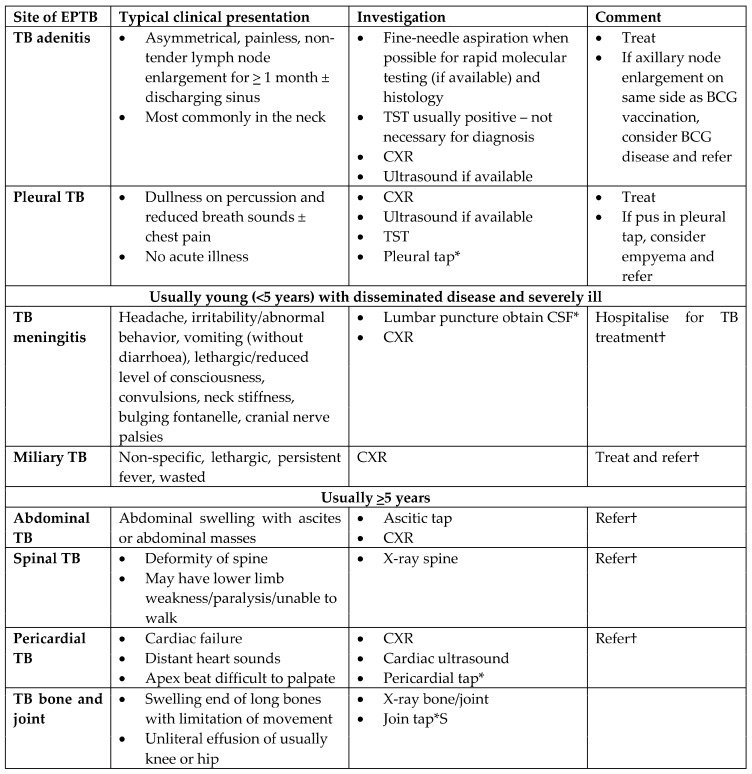
Typical clinical features of EPTB and suggested investigations, from ‘Diagnosis And Management of Tuberculosis In Children And Adolescents: A Desk Guide For Primary Health Care Workers’ [93]. * Typical findings: straw coloured fluid, exudate with lymphocytic predominance and high protein; sample could be sent for rapid molecular diagnostic testing and culture. † Referral may be necessary for investigation and laboratory support, as well as clinical care. If all options for referral have been explored and referral is not possible, start TB treatment. If TB meningitis is suspected, start TB treatment immediately with recommended regimen for TB meningitis.

EPTB is also common in children and may coexist with PTB [93]. TB lymphadenitis is the most common form of EPTB, while miliary TB and TBM are the most severe. Other forms of EPTB are also relatively common and include pleural TB, osteoarticular TB, spinal TB, pericardial TB, and abdominal TB. Presentation and symptoms vary with age and site of involvement. Figure 2 summarizes the clinical features of different forms of EPTB as well as the suggested investigations for each site of disease [93].

TB lymphadenitis of the cervical lymph nodes is the most common site of node involvement, and children generally present with a history of persistent (longer than one month) swelling that is not responsive to other treatments such as antibiotics. Clinical findings include non-tender, firm, enlarged cervical lymph nodes (especially if more than two by two centimeters) with or without fistula formation. Other clinical findings suggestive of EPTB include dullness to percussion and reduced breath sounds (pleural TB effusion); non-tender swollen joints with painful or abnormal gait (osteoarticular TB); distant or muffled heart sounds of new-onset heart failure (pericardial TB); distended abdomen with or without ascites (abdominal TB); and signs of meningitis, especially if not responding to antibiotic treatment [39]. It should be noted that TBM disproportionately affects young children, is associated with high mortality and morbidity, and is considered the most devastating form of TB. Recent modeling suggests that two-thirds of children with TBM die, while more than two-thirds of survivors suffer neurological sequalae [94]. The clinical presentation of TBM in children can be non-specific (including fever, headache, irritability, lethargy, poor feeding) or more specific (nuchal rigidity, focal neurological deficits, and seizures), making the differential broad and diagnosis difficult. Clinicians should always consider TB as a pathogen in suspected meningitis cases, especially in high TB prevalence settings, where it is estimated that TBM accounts for up to one-third to one-half of all bacterial meningitis cases [95]. TBM is considered a medical emergency, and urgent investigations (e.g., lumbar puncture, imaging) are warranted.

### 3.2. Tests for TB Infection

Tests for TBI evaluate the presence of an immune response to tuberculin antigens and include WHO-approved skin testing (e.g., TST) and IGRA [39]. In children with HIV, SAM, other severe illnesses, or contact with a person with TB, an induration of five mm or more on TST is considered positive. For children without these conditions (regardless of previous BCG vaccination status), an induration of 10 mm or more on TST indicates a positive result [39]. As noted previously, a positive result on TST does not distinguish between TBI and TB disease, and a negative test does not exclude TBI or TB disease. A positive result on a test for TBI is particularly useful when there is no known TB contact history. A positive test can be useful in supporting a diagnosis of PTB and/or EPTB in children with clinical features suggestive of TB who are bacteriologically negative or cannot provide an adequate specimen for laboratory testing.

### 3.3. Radiological Investigations

Radiological investigations, especially chest imaging, are important and useful tools to support the clinical diagnosis of TB in children, although each modality of chest imaging has specific benefits and limitations. Chest radiographs (CXRs) are currently the primary radiologic investigation used in children for the diagnosis and assessment of PTB, given relatively low-dose ionizing radiation and cost effectiveness despite the limitations of high inter- and intra-reader variability [96]. As most children will be diagnosed clinically, CXR can be helpful not only in supporting a decision to treat for TB but also in assessing severity of disease. CXR findings should not be used in isolation to make a TB diagnosis but should be interpreted in the context of clinical signs and symptoms, TB exposure history, and bacteriologic investigations, whenever feasible [39]. WHO has evaluated and recommended clinical decision algorithms that incorporate CXR and other available evidence to guide a decision to treat a child for TB (see below, Section 3.5).

Most children with pulmonary or intrathoracic TB will have an abnormal CXR suggestive of TB, but radiologic findings can be subtle and non-specific. As such, CXR in young children should be performed by a skilled radiographer and interpreted by a HCW trained to interpret radiographs in children. WHO recommends that full-size CXRs, including either posteroanterior or anteroposterior (for the youngest children), and lateral films be taken for children [39]. Lateral CXR is reported to be particularly helpful for imaging mediastinal lymph nodes and localizing lesions [97]. Additionally, there are insufficient data to guide CXR screening recommendations for TB in CLHIV; the current recommendation for CLHIV is for TB screening using symptoms, although evaluation of CXR as a screening tool in programmatic settings has been encouraged [14].

**Figure 3:**
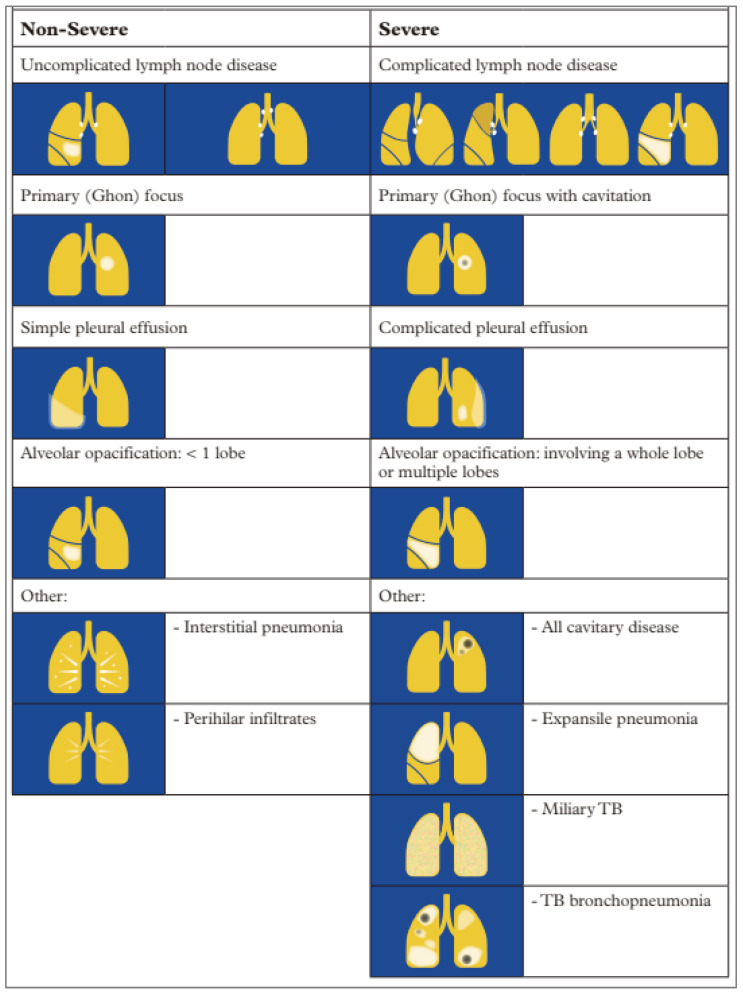
Common chest X-ray findings organized by disease severity, from the Union Diagnostic Chest X-Ray Atlas for TB in Children [97].

Unfortunately, both CXR access and experienced HCW confidence in interpreting CXR in children are limited in some high-burden, low-resource settings, reducing opportunities to increase case-finding among children. To provide a comprehensive guide to interpretation for radiographers and HCWs, The International Union Against TB and Lung Disease (The Union) released the second edition of the Diagnostic CXR Atlas for TB in Children in 2022 [97]. The Union Atlas provides comprehensive detail for assessing the technical quality of a film as well as interpreting radiologic changes and determining severity of disease by outlining a step-by-step process with example images showing common manifestations as shown in Figure 3, reproduced with permission [97].

#### 3.3.1. Common Manifestations of TB Disease on Chest X-ray

The most common radiological manifestations of PTB (including intrathoracic TB) in children are enlarged or perihilar lymph nodes, alveolar opacification, pleural or pericardial effusion, and miliary patterns consistent with disseminated TB [39,97]. Some features are more specific for TB than others; mediastinal lymph nodes, miliary patterns, pleural effusions, and cavitation are all specific to TB, and radiologic findings consistent with these features can be confidently used to make a TB diagnosis in children. HIV infection can complicate CXR interpretation for diagnosing TB; CLHIV are more likely to have miliary TB but may also present with residual lung scarring if they have had previous chest infections, and this scarring can be confused with TB [97]. It should be noted, however, that CLHIV stable on ART have CXRs more similar to children without HIV infection.

#### 3.3.2. Assessing TB Disease Severity

Determining the severity of TB disease on CXR allows for identification of patients who are at increased risk for poor outcomes and may require admission or referral to higher levels of care. In addition, severity of disease may determine a patient’s eligibility for new, short-course treatment regimens. In 2022, WHO recommended a shorter treatment regimen of four months for children with non-severe TB disease [14,39] (see below, Section 4). CXR plays a central role in determining eligibility for these shorter regimens in settings where CXR is available. The Union Atlas provides an overview of the features characteristic of non-severe and severe PTB in children (Figure 3) [97]. In general, the presence of airway compression, cavitation, miliary patterns, or more diffuse lung involvement indicates more severe TB disease.

#### 3.3.3. Computer-Aided Detection for CXR Interpretation

Computer-aided detection (CAD) has been recommended by WHO as a potential alternative to human interpretation of CXR when CXR is used as a screening tool for TB in adults and older adolescents; however, at the time of publication, more data are needed to validate the performance of CAD platforms for screening for TB in children [72]. For the reasons mentioned above, the sensitivity and specificity of CXR in screening for TB in younger children may differ from their performance in older adolescents or adults. The performance of CAD in pediatric populations for screening is an area of intense interest and research. A database, maintained by the Stop TB Partnership and AI for Health, lists commercially available CAD software with up-to-date information about certification and evidence for each product [98].

#### 3.3.4. Other Chest Imaging Modalities

Other diagnostic imaging modalities, such as computed tomography (CT), magnetic resonance imaging (MRI), and ultrasound (US), can enhance diagnostic potential and improve TB case detection in children [96]. Each of these modalities has benefits and limitations. CT scanning is considered the gold standard for imaging PTB in children. CT scans have been found to promote earlier and more sensitive detection of TB disease and specific disease manifestations compared with CXR. CT scans also have higher sensitivity for detecting node involvement, greater ability to differentiate TB from non-TB lymphadenopathy, can aid with surgical planning, and may be useful for monitoring disease complications and treatment response. However, CT scanning is expensive, has limited availability, requires expertise, may require contrast, and includes exposure to ionizing radiation (although low-dose protocols are now in use). MRI has sensitivity and specificity parameters comparable to those of CT and may be able to differentiate TB lymphadenopathy from reactive lymph nodes based on signal intensity and heterogeneity. However, MRI is also expensive, has limited availability, needs specific expertise, and may require sedation/anesthesia. Chest/thoracic US has the advantages of being performed bedside, free of ionizing radiation, and relatively inexpensive, but it also requires user expertise and specialized equipment. Chest US is increasingly being used to radiologically diagnose childhood pneumonia, but information is limited on its use for diagnosis of PTB in children. Available data suggest that chest US detected abnormalities, especially lymphadenopathy, pleural effusions, and pericardial and abdominal TB, more frequently than CXR, with higher inter- and intra-reader agreement, and was also able to assess for EPTB [96,99,100]. Therefore, US is a promising modality for detecting PTB in children, but further studies should evaluate the diagnostic accuracy of US against a gold standard (such as CT).

Finally, newer imaging techniques such as dynamic 4D CT, positron emission tomography (PET)/CT, and CAD software may improve the radiological accuracy of TB diagnosis in children and help guide intervention and treatment [96].

### 3.4. Bacteriological Investigations

#### 3.4.1. Importance of Child-Friendly Specimens

While many available TB diagnostic tests are less sensitive in children than adults, bacteriologic confirmation of TB is still important and should be sought whenever possible to confirm the diagnosis and ensure patient access to downstream drug resistance detection services for appropriate treatment regimen selection [14]. Considering the challenges of collecting sputum and other invasive respiratory specimen types, it is imperative to consider and utilize alternative child-friendly specimens [101,102]. Annexes 3 and 4 of the WHO operational handbook on TB in children and adolescents provide an overview of respiratory and non-respiratory specimens and collection methods that can be used to diagnose TB in children [39]. WHO-approved respiratory specimens for diagnosing PTB in children include spontaneously expectorated sputum, induced sputum, gastric aspirates, nasopharyngeal aspirates (NPA), and stool. Collection of specimens (especially induced sputum, gastric aspirate, and NPA) carries the risk of potential aerosolized transmission to HCWs, and this risk should be managed during collection [39]. Considerations for each specimen type are discussed below, with performance characteristics noted in Table 3; considerations regarding caregiver acceptability are noted in the WHO Operational Handbook [39].

- Expectorated sputum is a low-cost, non-invasive respiratory specimen in children with high caregiver acceptability. Unfortunately, expectorated sputum is usually not feasible for younger children, who are often unable to expectorate.
- Induced sputum is a feasible and relatively non-invasive respiratory specimen to collect from young children with moderate caregiver acceptability. However, it requires medical equipment, hypertonic saline, trained health care personnel, increased biosafety considerations, and electricity.
- Gastric aspirate is a relatively invasive respiratory specimen type for young children and may have lower caregiver acceptability. The procedure is optimally performed early in the morning in a health care facility by trained personnel [103]. Gastric aspirates have specific handling requirements, as timing of neutralization and specimen storage temperature may impact specimen quality [104]. Gastric aspirates, however, are relatively high-quality specimens that can contain greater amounts of mycobacteria when effectively collected, which can improve the chances of bacteriological confirmation.
- NPA respiratory specimen collection is feasible in young children and less invasive than gastric aspirate with moderate caregiver acceptability, but it does require medical equipment and trained personnel and has lower diagnostic performance compared with other traditional respiratory sample types [105,106,107].
- Collection of stool specimens is a relatively new, low-cost, and non-invasive approach to obtaining (swallowed) respiratory specimens in children, including young children, and has high caregiver acceptability. Stool specimens do require additional laboratory processing and personnel training. Stool specimens have demonstrated increasing sensitivity with disease severity compared to gastric aspirates in some circumstances [86], although generally lower (Table 3). There are several methods for collecting and processing stool specimens. The Simple One-Step (SOS) stool processing method has been scaled most widely and has augmented the implementation of stool as a child-friendly, non-respiratory specimen for TB diagnosis [108]. The SOS method of processing stool has resulted in improved detection of TB compared to sputum specimens in CLHIV [109,110].

Other, non-respiratory specimens helpful for the diagnosis of TB in children include cerebrospinal fluid (CSF), serosal fluids and tissues, urine, fine needle aspiration biopsies, bone marrow, and blood for those living with HIV with signs and symptoms of disseminated TB [14,39].

The choice of which specimen or combination of specimens to utilize in a diagnostic evaluation will depend on the type of presumed TB disease (PTB, EPTB, or both), advantages and disadvantages of each specimen type (e.g., feasibility of collection in young children, invasiveness, laboratory requirements, and capabilities), and acceptability to the child and caregiver [39,81,111]. Whenever possible, multiple specimens of different types should be collected as quicky as possible to maximize diagnostic yield. In addition, each specimen type may have specific indications or approval for various laboratory tests and diagnostic instruments. For example, current WHO guidelines recommend certain specimen types for the diagnosis of PTB in children and adolescents using Xpert or Xpert Ultra: sputum (expectorated or induced), gastric aspirate, NPA, and stool [14,39]. Other WHO-recommended rapid diagnostics (WRDs) using respiratory specimens have only been validated on sputum. Additional information about indications and approvals for specimen types as well as performance of various sample types on WRD is discussed in more detail below and in Table 3.

#### 3.4.2. Culture for Mycobacterium Tuberculosis

Mycobacterial liquid culture for sputum and other approved specimen types is a highly sensitive test for TB diagnosis, with a lower limit of detection than molecular tests, which can improve rates of bacteriological confirmation among children who often produce specimens with very low and variable amounts of mycobacteria [112]. Although culture remains the gold standard for confirmation of TB and drug susceptibility testing, culture takes more time to produce a result than molecular tests (weeks instead of hours), is much more complex than molecular and urine-based lateral flow testing, requires specialized laboratory facilities with increased biosafety infrastructure, and requires enhanced personal protective equipment and biosafety testing practices for testers [113]. Culture of stool [114] for TB detection in children is not recommended. Although several countries continue to use smear microscopy for bacteriologic identification of TB—as it is a relatively simple, low-cost test—the diagnostic sensitivity and yield in children are incredibly low. A meta-analysis including studies with a total of 18,316 children reported that the sensitivity of smear microscopy was just 7% in children compared to 52% in adults [115]. Therefore, WHO recommends other diagnostic tests be used to confirm a diagnosis of TB in children [14].

#### 3.4.3. WHO-Recommended Rapid Diagnostic Testing (WRD)

WHO recommends the use of WRDs with varying ranges of pediatric specimen types (e.g., sputum, gastric aspirate, NPA, stool) to aid in the diagnosis of TB in children [14]. These tests are recommended instead of traditional smear microscopy and culture-based methods as they provide specific results within minutes or hours (as opposed to days or weeks for culture), several may be placed at point-of-care (POC) or near POC to improve patient access to testing services, and many provide simultaneous or quick reflex-based drug resistance results to guide appropriate treatment of children with TB. WHO recently consolidated these WRDs into classes based on their complexity and level of automation, which are reviewed accordingly below [14].

##### Xpert MTB/RIF, Xpert MTB/RIF Ultra

Xpert and Xpert Ultra, defined as molecular WRDs (mWRDs), are automated nucleic acid amplification tests run on GeneXpert instruments. The presence of Mycobacterium tuberculosis complex and rifampicin resistance are detected in less than two hours on both tests. Xpert changed the TB testing landscape when it became commercially available in 2010, and sensitivity was further improved with the introduction of Xpert Ultra in 2017 [116]. The additional targets for polymerase chain reaction (PCR) amplification and the larger chamber for DNA amplification used in Xpert Ultra reduced the limit of TB detection eight-fold, thereby increasing sensitivity [117], which is significantly higher than Xpert in CLHIV or children with smear-negative PTB [118,119]. Noting the shorter run-time, higher sensitivity, and lower limit of detection of Xpert Ultra, a transition from Xpert to Xpert Ultra has been recommended [106,120].

WHO recommends the use of Xpert and Xpert Ultra to test sputum (including induced sputum, gastric aspirate, and NPA), stool, and lymph node biopsy, among other specimens [14,39]. The diagnostic performance of each of these specimens using various tests is important when considering which specimen(s) to collect for diagnostic evaluation of a child. Table 3 summarizes key performance characteristics of various child-friendly sample types on currently recommended tests.

As discussed above, due to the low bacillary burden and quantity of specimens that can be obtained from children, collecting multiple specimens, either of the same or different types, has become more common in recent years. This approach was found to increase the microbiological yield using Xpert, Xpert Ultra, or culture [81,111]. Combining the results of testing one NPA and one induced sputum specimen using Xpert Ultra increased the sensitivity to 80%, compared to 46% using one NPA and 74% using one induced sputum specimen [121]. In instances where induced sputum is not feasible, testing two NPA specimens using Xpert was found to have a greater diagnostic yield [105]. On culture or Xpert, NPA in combination with another NPA or a stool specimen yielded sensitivity and specificity comparable with induced sputum or gastric aspirate, which are more invasive and can be difficult to obtain. The enhanced diagnostic yield of combining NPA and stool specimens was also confirmed in CLHIV [122]. Using Xpert Ultra, stool demonstrated equivalent sensitivity compared to the more invasive gastric aspirate specimen type in a recent study in China [86].

##### Truenat MTB, MTB Plus, and MTB-RIF Dx

Truenat is another automated nucleic acid amplification-based system and mWRD that includes Truenat MTB, Truenat MTB Plus, and Truenat MTB-RIF Dx assays. The tests are run using a near-POC dual-instrument system for the detection of MTB and reflex-based detection of rifampicin resistance. A recent study in India demonstrated that when using pediatric specimens including gastric aspirate, induced sputum, and bronchoalveolar lavage, sensitivity and specificity were comparable to the results obtained using Xpert assays [87].

##### TB-LAMP

TB loop-mediated isothermal amplification (TB-LAMP) is a mWRD that is relatively simple to perform using sputum and requires minimal laboratory infrastructure, enabling its use in more peripheral settings [14,123]. While the TB-LAMP assay has been shown to have a higher sensitivity when compared with smear microscopy and comparable sensitivity to Xpert [88,89], it cannot be used with other child-friendly sample types, does not test for drug resistance, and requires electricity to perform.

##### Lateral Flow Lipoarabinomannan (LF-LAM)

The WRD lateral flow lipoarabinomannan (LF-LAM) assay utilizes urine for detection of mycobacteria and is recommended for use in children, adolescents, and adults with presumptive TB who are co-infected with HIV [14,73,124]. LF-LAM detects a cell wall component present in several mycobacterial species that is secreted in the urine of PLHIV with disseminated TB, including those with severe illness or AHD [125]. LF-LAM has substantial benefits as an initial test for TB diagnosis in CLHIV, allowing rapid, non-invasive bacteriological confirmation of disease and rapid TB treatment for severely ill children and those with AHD (including all HIV-infected children under five years of age).

Currently, the only LF-LAM test available on the market is the Abbott Determine TB LAM Ag assay. This test has a reported sensitivity range of 16% to 52% among symptomatic children with severe malnutrition [126]. The current version of the Abbott Determine TB LAM Ag test does not meet target product profile standards for sensitivity set by WHO [127] and cannot detect drug resistance. However, its use in adult and adolescent PLHIV has resulted in widespread improved patient access to TB diagnostic testing services and increased TB case finding yields—particularly among those who share barriers to testing with pediatric patients, such as being unable to produce sputum or producing paucibacillary sputa. In addition, it is the only TB diagnostic test with a proven morbidity and mortality reduction benefit among PLHIV 18 years and older. These benefits may also apply to pediatric populations, for which programmatic and operational research evaluations are underway. Research and development on multiple next generation LF-LAM tests aimed at improving test performance in HIV-positive and HIV-negative populations is ongoing, with multiple controlled laboratory and field evaluations underway.

##### C-Reactive Protein Testing

C-reactive protein (CRP) is a molecule secreted by the liver in response to inflammation. CRP tests quantify the amount of this molecule in finger-stick or venous blood as a non-specific indicator of immune health. Tests can be conducted at POC or in peripheral settings and have the potential to provide rapid results (two to three minutes), depending on the assay and instrument used. WHO has recommended CRP testing as a screening tool for TB in children and adolescents living with HIV 10 years of age and older as part of screening algorithms [72]. In non-immunocompromised, clinically or bacteriologically confirmed TB-positive pediatric patients in India, the median CRP was found to be 25 mg/L compared to 0.54 mg/L in the healthy control group, making this test a potentially useful TB ‘rule out’ test, especially for older children and adolescents [128]. However, recent studies suggest that CRP performance in children under 10 years of age did not meet the requirements for a TB triage test [129]; therefore, further research is needed to understand the utility and programmatic performance of CRP for TB screening in HIV-infected children [128].

### 3.5. Treatment Decision Algorithms

Bacteriologic confirmation of TB in children is important whenever possible but may not always be feasible. A decision to start TB treatment based on clinical findings should not be delayed if a child is bacteriologically negative or if other investigations are unavailable, particularly in children with presumed TBM, HIV, SAM, or other conditions that increase the risk of developing severe disease. However, when the clinical diagnosis is uncertain, it is important to recognize that a treatment decision does not always have to be made based on initial presentation. Many children with TB or presumptive TB are not severely unwell, and so a follow-up evaluation in two to three weeks with reassessment of clinical features and weight can be very helpful in treatment decision-making, including to avoid overdiagnosis and treatment [18,39]. Clinical or treatment decision algorithms have been used for decades to integrate available information to inform a decision, regardless of access to bacteriologic confirmation. They weigh relevant signs and symptoms, clinical assessment, and TB exposure history, as well as findings from relevant investigations, and in some cases, they also stratify populations based on risk factors with variable thresholds for deciding to treat. Such algorithms allow for some standardization of the clinical decision-making process, weighing key factors and providing a score for informing a treatment decision. These algorithms are invaluable in peripheral settings with limited diagnostic capabilities and/or pediatric TB expertise; however, many such algorithms have not been validated and are instead based on expert opinion and availability of various diagnostic capabilities within a given country or setting.

WHO included a generic, interim recommendation for the use of treatment decision algorithms in its new guidelines [14]. This recommendation was based on an analysis of seven existing clinical decision algorithms developed between 1987 and 2019 [24,130,131,132,133,134,135] compared with the ‘standard of care’ algorithm from The Union’s 2016 Desk Guide for Management of TB in Children and Adolescents [136], which was based on the most recent WHO diagnostic guidelines at the time. The performance of each algorithm was tested against an individual patient data meta-cohort of children under 10 years of age representing a range of geographic regions, comorbidities, and demographics. Performance of the algorithms ranged from 16% to 95% sensitivity and 9% to 89% specificity, with none considered optimal. Performance among key subpopulations in the meta-cohort also varied: CLHIV: sensitivity (24–92%), specificity (15–87%); SAM: sensitivity (33–93%), specificity (10–88%); and infants (<12 months of age): sensitivity (17–93%) and specificity (13–86%) [14]. On balance, algorithms with higher sensitivity were favored over those with higher specificity, considering the likelihood of a poor outcome if a TB diagnosis is missed in this population.

**Figure 4:**
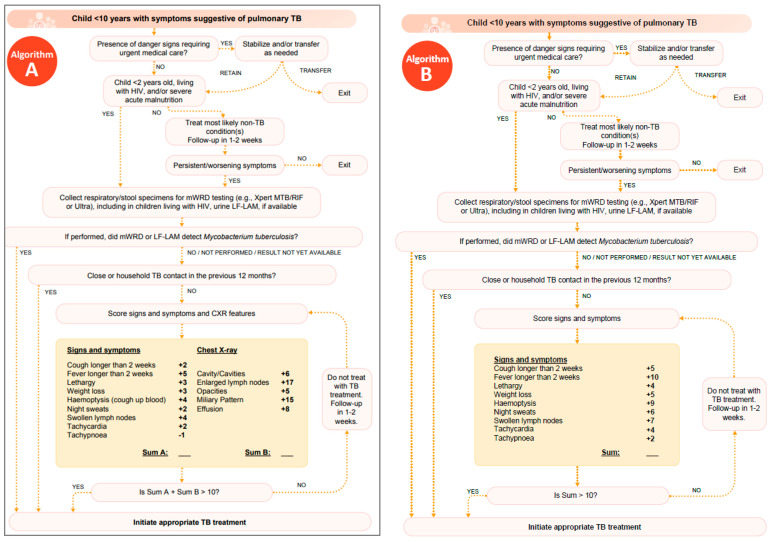
(**A**). The WHO-recommended integrated treatment decision algorithm for use in settings with access to CXR, from the WHO operational handbook on tuberculosis. Module 5: management of tuberculosis in children and adolescents; (**B**). The WHO-recommended integrated treatment decision algorithm for use in settings without access to CXR from the WHO operational handbook on tuberculosis. Module 5: management of tuberculosis in children and adolescents [39].

Following this analysis of existing algorithms, the WHO Guideline Development Group formulated new integrated algorithms for use in settings with CXR (Algorithm A) or without CXR (Algorithm B) (Figure 4A,B) with the best balance of performance: sensitivity of approximately 85% and specificity between 30% and 37% [39]. While a score and decision can be derived from these algorithms in the absence of bacteriologic testing, the guidelines emphasize that bacteriologic confirmation should be sought whenever feasible. These algorithms include an initial stratification of children with danger signs who require stabilization and/or referral to a higher level of care. Danger signs are specified by age group in the operational handbook and include inability to eat or drink, severe pallor, stridor, obstructed breathing, and seizures, among others [39]. This stratification is followed by a second: children who are at high risk of progression to disease (and require immediate diagnostic investigations for TB) and those who are not at high risk. In the absence of bacteriologic confirmation, each algorithm provides weighted scoring for signs and symptoms (Algorithms A + B) and CXR findings (Algorithm A only). WHO has emphasized the need to validate and evaluate these algorithms in routine program settings as well as develop evidence-based algorithms for specific subgroups at high risk of rapid progression to TB disease: infants, CLHIV, and children with SAM. WHO, in collaboration with external investigators, is evaluating these algorithms in various clinical settings with the aim of reaffirming and/or revising this recommendation following a review of additional evidence. WHO’s interim recommendation expired 24 months after the guideline release (March 2024). No formal reaffirmation has been issued while WHO awaits reviews of evidence of performance characteristics for treatment decision algorithms in various settings and populations. Updated guidance is expected after reviews of evidence are completed, likely in 2025.

## 4. Treating a Child for Drug-Susceptible TB Disease

Children generally respond very well to treatment and have better treatment outcomes than adults, and research over the past 10 years has led to notable advances in the treatment of both DS and DR TB in children. Prior to 2022, the standard recommended regimen for children with DS TB (all forms except TBM and osteoarticular TB) was a six-month course of treatment with a two-month intensive (initial) phase (daily isoniazid [H], rifampicin [R], and pyrazinamide [Z] with or without ethambutol [E]) and a four-month continuation phase (daily HR). Ethambutol is recommended in the intensive phase in patients whose TB is multibacillary (such as smear-positive or high detection on WRD) and is also included in regimens for all TB cases in settings with high HIV prevalence or isoniazid resistance [137]. This regimen is commonly abbreviated: 2HRZ(E)/4HR. Longer, multidrug treatment, such as the 2HRZ(E)/4HR regimen, has been the standard for drug-susceptible TB for decades because of fundamental characteristics of bacilli growth and heritable drug resistance; the likelihood of drug resistance increases with increased bacilli load (a component of the determination of disease severity) [138]. Following the release of findings from trials evaluating shorter treatment regimens for DS TB disease among children (see Section 4.1), WHO now recommends different regimens depending on severity of disease, age, site of disease, and other factors, which are outlined below We refer readers to other resources for discussion of the treatment of DR-TB in children. Medication used for treatment of DS TB, regardless of length of treatment, should be dosed based on patient weight. Weight-based dosage guidance can be found in the WHO operational handbook companion to the 2022 guidelines [39]. Table 2 summarizes composition and implementation considerations for the regimens described below.

### 4.1. Non-Severe Drug-Susceptible TB Disease (Shorter Treatment Regimen)

Shorter treatment regimens for children and adolescents with TB disease have the potential to improve adherence, reduce the risk of adverse events, and lower costs for patients, families, and health care systems. Accordingly, the Shorter Treatment for Minimal Tuberculosis in Children (SHINE) trial evaluated the suitability of a shorter course of treatment for children and adolescents with non-severe DS TB [32]. This multicenter randomized controlled trial compared the effectiveness of a shortened four-month regimen of treatment (2HRZ/2HR or 2HRZE/2HR) with the previous standard six-month regimen (2HRZ/4HR or 2HRZE/4HR) in children under 16 years of age with non-severe TB. Non-severe TB was defined as peripheral lymph node TB (also referred to as TB lymphadenitis) or PTB confined to one lobe of the lung without cavitation, airway obstruction, or miliary disease. The SHINE trial found that patients treated with the shorter four-month regimen had non-inferior outcomes to those treated with the standard six-month regimen. Of note, children and adolescents living with HIV were included in the SHINE trial and were also found to have non-inferior outcomes with a four-month treatment regimen that included ethambutol in the intensive treatment phase. In 2022, WHO recommended the four-month regimen for patients from three months through 16 years of age with non-severe TB based on these results [14,39].

One important consideration in implementing this new recommendation is the ability to differentiate non-severe from severe TB. In the SHINE trial, participants were classified as having ‘non-severe’ TB disease based largely on radiographic findings (Figure 3). WHO provides additional guidance on implementing shorter treatment regimens for children in settings with variable diagnostic capabilities, including settings where CXR and diagnostic testing are both available, where only diagnostic testing is available, and where neither is available, although eligibility is consequently limited depending on these capabilities [39]. Given such diagnostic requirements, the implementation of shorter course treatment regimens should consider existing national guidance, available resources, and diagnostic capabilities [39].

### 4.2. Severe Drug-Susceptible TB Disease

WHO recommends that any child not meeting eligibility criteria for the shorter treatment regimen for non-severe disease (2HRZ(E)/2HR) should be treated with the six-month regimen (2HRZ(E)/4HR) unless there is a clinical indication for longer regimen. Clinical presentations consistent with severe pulmonary TB disease include complicated pleural effusion, cavitary lung disease, and bilateral lung involvement. Most forms of EPTB (Figure 2) are classified as severe disease requiring treatment with the six-month regimen, except for TBM and osteoarticular TB, which are treated differently (see below) [14,39]. In addition, if a determination of severity cannot be made, the six-month regimen remains the recommended default regimen. 

### 4.3. Drug-Susceptible Pulmonary TB Disease (Children ≥ 12 Years)

Children ≥ 12 years or age and >40 kg are also eligible for an alternative shorter regimen that is recommended for drug-susceptible pulmonary TB, regardless of severity. Recently, a multi-site randomized, controlled non-inferiority trial compared two alternative, shorter treatment regimens to the former standard six-month regimen (2HRZ(E)/4HR) among persons ≥12 years [139]. This trial found that one regimen, the four-month, daily regimen including isoniazid, rifapentine, pyrazinamide, and moxifloxacin, was non-inferior to the six-month regimen. This regimen (2HPMZ/2HPM) includes two months of daily isoniazid, rifapentine, moxifloxacin, and pyrazinamide (2HPMZ), followed by 9 weeks of daily isoniazid, rifapentine, and moxifloxacin (2HPM). These highly potent medications accelerate culture conversion and effectively shorten the length of treatment required to achieve cure [139]. WHO now recommends this regimen for all forms of drug-susceptible pulmonary TB for people over the age of 12 years, consistent with the study population [14,140]. All medications in this regimen are available in pediatric formulations but not in fixed-dose combinations, likely resulting in a higher pill burden. In addition, DST to confirm susceptibilities may be challenging to implement in high-burden settings. Medications in the regimen may be more costly than the standard and alternate four-month regimen for children (above) [14,39,140].

### 4.4. Severe Drug-Susceptible TB Disease: TB Meningitis and Osteoarticular TB

As previously noted, TBM predominately affects children under four years of age and is associated with significant morbidity and mortality [141,142]. Since 2010, the standard treatment regimen recommended by WHO has been a 12-month treatment regimen (2HRZE/10HR). However, the standard treatment regimen utilized for TBM in South Africa, a high-TB burden country, has been six months of intensive therapy with HRZ plus and ethionamide (Eto) (denoted 6HRZEto), with higher doses of isoniazid and rifampicin compared with the WHO-recommended standard 12-month regimen [143] (see Table 2).

A meta-analysis comparing the standard 12-month regimen with the intensive six-month regimen for treatment of TBM found that outcomes were similar between the two regimens, leading to a conditional recommendation for the six-month intensive regimen as an alternative to the standard 12-month regimen [14]. As there are limited data available on patients living with HIV and TBM, it is recommended by WHO that CLHIV with TBM receive the standard 12-month treatment regimen. Additionally, WHO recommends that patients with TBM be hospitalized for treatment initiation and monitoring if possible.

Osteoarticular TB is similarly associated with significant morbidity and mortality in young children, but is relatively rare, comprising less than 6% of extrapulmonary TB in children [144]. WHO recommends that osteoarticular TB also be treated with a 12-month treatment regimen (2HRZE/10HR) [14].

### 4.5. Drug-Resistant TB Disease

Discussion of management strategies and considerations for DR TB is beyond the scope of this article but is discussed in a separate article in this Special Issue [145].

### 4.6. Treatment Support and Monitoring

TPT and TB treatment response assessments should focus on the following: (1) weight check (for response to treatment and to guide appropriate drug dosage); (2) resolution, persistence, or development of new TB symptoms (i.e., cough, fever, poor weight gain, and reduced playfulness); (3) presence of adverse events related to the drug regimen (e.g., check for signs and symptoms of hepatitis and other relevant adverse events); and (4) adherence to treatment (verbal discussion and/or pill counts), which can be especially challenging in children [39] (Table 2). Routine laboratory monitoring is not necessary for healthy children and adolescents but should be considered for those with immunocompromise, existing hepatic disease, or taking hepatotoxic medications.

Children and adolescents have unique health care and social needs, and adherence to TPT and TB disease treatment remains a challenge, although progress has been made to develop approaches such as decentralized care and digital tools to make treatment more patient-friendly and improve treatment outcomes [23,78]. All-oral and shorter drug regimens in child-friendly drug formulations can also help improve adherence and treatment completion rates [76,146]. Potential barriers to adherence include lack of child-friendly drug formulations, lack of conviction about the utility of treating TBI, not having one or more caregivers, changes in routine for the child (e.g., school holidays), and issues related to stigma [39,146].

Another important intervention that could be implemented, if resources permit, is providing nutritional support to both the child and their family, especially if the index case is a household member. In addition to improving treatment outcomes, a recent randomized trial that evaluated the effect of nutritional support on TB incidence in household members found that providing nutritional support was associated with reductions of 39% to 48% in TB incidence in intervention households during a 2-year follow-up period [147].

### 4.7. Child-Friendly Formulations

All first-line medications used for treatment of DS TB are available in child-friendly, water-dispersible formulations that allow for more precise medication dosing and avoid the problems associated with crushing, splitting, or inaccurately measuring adult versions of the same drugs. There has also been progress in the development of child-friendly formulations for DR TB medications; currently, 14 quality-assured DR TB medicines are available [31]. In addition, WHO recommends the use of FDC medication formulations that combine isoniazid, rifampicin, and pyrazinamide (for the intensive phase of treatment) or isoniazid and rifampicin (for the continuation phase of treatment) [14]. Ethambutol is also available in a child-friendly formulation when indicated to complete a regimen. The use of these FDC tablets reduces the likelihood of prescription errors, results in a lower pill burden on the child and their families and improves the likelihood of reaching therapeutic concentrations. Child-friendly/dispersible FDCs are readily available through Stop TBs Global Drug Facility (GDF) for high-incidence countries and should be prioritized by national TB programs.

## 5. Looking Forward

The past decade has been one of accelerated innovation on all fronts for TB, including in the area of childhood TB. With new innovations and refinements of existing approaches, health care providers have more tools available than ever before to manage TB in children. Much can be achieved to close the current gaps in detection, treatment, and prevention by applying existing tools and policies. We have summarized common challenges, misperceptions, and available solutions to address these in Table 4.

Despite substantial progress, gaps in TB care for children still exist, including in both implementation and research, although efforts continue to tackle and address these gaps. A recent monograph focuses on some of the key gaps and implementation challenges in TB care for children and adolescents, outlines the critical role of multisectoral partnerships, and discusses and provides examples of facilitating integration of innovations into routine programs [148].

Research and development specifically for children has led to an increasingly rich pipeline of new tools and methods to address gaps in TB prevention, diagnosis, and treatment, as well as rich data from recently developed collaborative research platforms and multinational trials that evaluate impact in a range of diverse settings (e.g., SHINE [32], TB-Speed [149], RaPaed-TB [150], and TB CONTACT [151]). Also of particular note are research efforts related to TB vaccine candidates and novel specimen types to aid in TB diagnosis. In the past decade, 11 new candidate TB vaccines have been evaluated in pediatric vaccine trials [147]. There are nine currently active pediatric TB vaccine trials; Cranmer et al. summarize these trials according to trial phase, age group, and primary endpoint [152,153,154]. In addition, promising potential specimen types include oral or tongue swabs as well as face mask sampling to detect TB. Oral or tongue swabs have shown wide-ranging sensitivity compared to sputum specimens when tested using Xpert [155,156,157] or Xpert Ultra [158], but hold enormous promise in increasing diagnostic yield among children. Similarly, face mask sampling arose during the COVID-19 pandemic as a potential easy-to-obtain specimen for testing [159,160,161]. Although research has thus far focused on adults, several studies are underway evaluating its utility in pediatric populations. Continued investment in TB research and development, specifically for children, will be important to develop better tools to overcome remaining challenges.

This manuscript summarizes existing guidance and best practices and identifies aids, tools, and additional resources to assist clinicians and health care providers in optimizing access to and care for children with TB. It may also inform further study on translation of policy into practice to improve prevention and management of pediatric TB. By understanding the unique vulnerabilities and special considerations for children with TB and applying evidence-based tools and approaches to overcome them, health care providers can help reduce the untenable burden of disease and death among children.

## Figures and Tables

**Table 1:** Selected resources, guidelines, and training materials for management of TB in children and adolescents, adapted and informed by the Roadmap to end TB in children and adolescents [13].

Resource	Description	Link (accessed on 26 October 2023)
**Comprehensive Resources and Guidance**		
WHO Consolidated Guidelines on Tuberculosis: Module 5: Management of Tuberculosis in Children and Adolescents (2022)	Consolidated guidelines of new and existing recommendations (>130 recommendations) on TB screening, prevention, diagnosis, treatment, and models of care for children and adolescents	https://www.who.int/publications/i/item/9789240046764
WHO Operational Handbook on Tuberculosis: Module 5: Management of Tuberculosis in Children and Adolescents (2022)	Detailed practical considerations for implementing recommendations in the WHO guidelines; provides treatment regimen composition and dosing charts, adverse events mapping, costing information and monitoring and evaluation templates for specific activities along with other tools and resources	https://www.who.int/publications/i/item/9789240046832
Diagnosis and Management of Tuberculosis in Children and Adolescents: A Desk Guide for Primary Health Care Workers (4th Edition), International Union Against Tuberculosis and Lung Disease (2023)	A decision-making aid intended for use by primary health care workers; the fourth edition includes revisions to diagnostic approaches and options for treatment of both TB infection and disease and is available in African and Asian editions, which incorporate more region-specific examples	https://theunion.org/technical-publications/diagnosis-and-management-of-tuberculosis-in-children-and-adolescents-a-desk-guide-for-primary-health-care-workers
WHO Global TB Report (2023) (updated annually)	Provides a comprehensive and up-to-date assessment of the TB epidemic and of progress in prevention, diagnosis, and treatment of the disease at global, regional, and country levels	https://www.who.int/publications/i/item/9789240083851
WHO Information Sheet: Management of Tuberculosis in Children and Adolescents (2022)	Provides a high-level overview of key concepts, burden of disease, and program and policy priorities	https://www.who.int/publications/m/item/information-sheet-management-of-tuberculosis-in-children-and-adolescents
**Trainings**		
WHO e-course for health care workers on the management of tuberculosis in children and adolescents (2023)	Online, self-paced e-course containing teaching modules on epidemiology, diagnosis, treatment and prevention of TB and drug-resistant TB with a focus on latest guidelines; developed by WHO and The Union with support from CDC	https://openwho.org/channels/end-tb
WHO e-course on tuberculosis in children and adolescents: programmatic considerations (2023)	Online, self-paced e-course containing teaching modules with programmatic considerations for managing TB in children and adolescents; developed by WHO with support from CDC	https://openwho.org/channels/end-tb
The Union e-course on child and adolescent TB for health care workers (Updated 2022)	Online, self-paced e-course with comprehensive learning modules on epidemiology, detection, treatment and prevention of TB and drug-resistant TB; updated to align with 2022 WHO guidelines on management of TB in children and adolescents; developed by The Union in collaboration with the Elizabeth Glaser Pediatric AIDS Foundation	https://theunion.org/our-work/union-courses
The Union e-course on CXR interpretation in children with presumptive TB (2023)	Online e-course companion to the Union Atlas reviewing the Atlas’ algorithmic approach to CXR interpretation; includes learning modules with reviews of various findings on CXR among children with presumptive TB	https://coursesonline.theunion.org/theunion/2023/interpretation-of-chest-x-rays-in-children-with-tb/393947/
TB-SPEED CXR training to diagnose childhood TB	TB-Speed is a Unitaid funded project aimed at reducing childhood TB mortality by developing decentralized, cost-effective, and feasible diagnostic strategies to increase case-finding in children; presentation-based training covers common features and findings on CXR in children with pulmonary TB	https://www.tb-speed.com/wp-content/uploads/2020/09/TB-Speed_Interpret-Child-CHR.pdf
**Diagnosis and Treatment**		
Making the best out of available tools and approaches: Summary guidance for microbiological and clinical diagnosis of pulmonary TB among children	An information note describing the complementary diagnostic approaches of microbiological testing and clinical evaluation and assessment; developed by the Pediatric TB Operational and Sustainability Expertise Exchange Taskforce of the Child and Adolescent TB Technical Working Group	https://www.stoptb.org/file/16091/download
WHO Global Laboratory Initiative: Practical manual for processing stool samples for diagnosis of childhood TB	Outlines practical guidance for collecting and processing stool samples for use with Xpert and Xpert Ultra; developed by the Global Laboratory Initiative,	https://www.who.int/publications/i/item/9789240042650
The KNCV Simple-One-Step Method Stoolbox: An implementation package for the SOS Stool method to detect TB and rifampicin resistance	A guide and toolkit outlining how to implement the simple-one-step stool processing method for Xpert and Xpert Ultra; developed by the KNCV Tuberculosis Foundation	https://www.kncvtbc.org/en/sos-stoolbox/
Diagnostic CXR Atlas for Tuberculosis in Children: A Guide to Chest X-Ray Interpretation, International Union Against Tuberculosis and Lung Disease (2nd Edition, 2022)	Outlines an algorithmic approach to evaluating CXRs in children and integrating these findings into treatment decisions based on classification of CXR features by radiological severity; presents numerous CXR images with detailed annotations	https://theunion.org/technical-publications/diagnostic-cxr-atlas-for-tuberculosis-in-children
Diagnostic CXR Atlas for Tuberculosis in Children: Image Library, International Union Against Tuberculosis and Lung Disease (2022)	A companion to the Union Atlas containing a collection of CXR images from children under 15 years of age who present with symptoms and signs of TB; CXRs in this library provide examples of features highlighted in the Union Atlas arranged into seven categories: uncomplicated lymph node disease, cavitary disease, complicated lymph node disease, consolidations, miliary TB, pleural effusions and other	https://theunion.org/technical-publications/diagnostic-cxr-atlas-for-tuberculosis-in-children
Clinical standards for Drug-Susceptible TB in Children and Adolescents. (2023)	Consensus clinical standards designed to provide guidance on ‘best practice’ for diagnosis, treatment and management of drug-susceptible pulmonary TB	https://pubmed.ncbi.nlm.nih.gov/35768923/
Management of Multidrug-Resistant Tuberculosis in Children: A Field Guide, Sentinel Project for Pediatric Drug-Resistant TB (5th Edition, 2022)	Provides information on clinical and programmatic practice for managing MDR-TB in childrenes; includes case examples to demonstrate how recommendations can be put into practice; complements existing recommendations	https://sentinel-project.org/wp-content/uploads/2022/03/DRTB-Field-Guide-2021_v5.pdf
**Prevention**		
WHO operational handbook on tuberculosis: module 1: prevention: tuberculosis preventive treatment	Companion implementation guide to the 2020 WHO guidelines on TPT, providing practical guidance on implementing TPT	https://www.who.int/publications/i/item/9789240002906
Tuberculosis Infection in Children and Adolescents: Testing and Treatment: Clinical Report and Guidance from the American Academy of Pediatrics (2021)	Guidance from the American Academy of Pediatrics and its Committee on Infectious Diseases; includes effective approaches to the testing and treatment of TBI in children and adolescents	http://publications.aap.org/pediatrics/article-pdf/148/6/e2021054663/1354238/peds_2021054663.pdf
PEPFAR TPT Implementation Guide and Toolkit	Provides guidance and tools for ministries of health to develop comprehensive TPT programs for PLHIV, including children; includes considerations for incorporating TPT into differentiated service delivery models for adults and children	https://www.state.gov/pepfar-solutions/resources-and-tools/tb-preventive-treatment-tpt-implementation-tools/
**Special Issues and Key Publications**		
Pathogens Special Issue: Recent Advances and Ongoing Challenges in the Management of Tuberculosis in Children and Adolescents (2022)	Peer-reviewed “state-of-the-art” articles by recognized leaders in child TB research and clinical management, as well as perspectives on challenges for programmatic implementation in “real life” settings; addresses topics such as epidemiology, clinical care, prevention, and health systems strengthening for DS and DR TB in children and adolescents	https://www.mdpi.com/journal/pathogens/special_issues/Tuberculosis_Children_Adolescents
Journal of the Pediatric Infectious Diseases Society, Special Supplement: What’s New in Childhood Tuberculosis? (2022)	Experts in childhood TB address several aspects of the TB epidemic in children, including TB/HIV, COVID-19, medication interactions and adherence, diagnostics, new treatment regimens, vaccine development, and programmatic approaches to TB care and services	https://academic.oup.com/jpids/issue/11/Supplement_3
**Advocacy and Program**		
WHO Roadmap for ending TB in children and adolescents (3rd Ed, 2023)	Presents important data and tools which can be used to consolidate and advance advocacy, commitment, resource mobilization and joint efforts by all stakeholders to provide health care and address the burden of TB among children	https://www.who.int/publications/i/item/9789240084254
The Union/CDC Sub-Saharan Africa Regional Child and Adolescent TB Virtual Centre of Excellence	A virtual network of public health experts in child and adolescent TB in the sub-Sahara Africa region, providing a community of learning and practice for child and adolescent TB; resources, training materials, webinars, and case discussions available on the website	https://theunion.us9.list-manage.com/track/click?u=6bdc29f3fb65cf617f7e060fa&id=07d0b79a2f&e=f06cc95456
Checklist of and budgeting tools for including child and adolescent interventions in Global Fund Against AIDS, TB, and Malaria applications for TB programs	Key activities and interventions related to the prevention and management of tuberculosis in children and adolescents that should be considered for inclusion in country proposals to strengthen programming and help closing the persistent policy-practice gap for child and adolescent TB	https://www.stoptb.org/checklist-tb-interventions-gf-proposals https://stoptb.org/wg/dots_expansion/childhoodtb/posee.asp

**Table 2:** Regimens for treating pediatric TB infection and drug-susceptible TB (non-severe, severe, and meningitis and osteoarticular).

Regimen	Drugs *	Interval of Administration	Duration (Months)	Monitoring Considerations **	Preferred Regimen	Considerations for Use
TB Infection						
3HR ^‡^	Isoniazid (H) Rifampicin (R)	Daily	3	Evaluate monthly For CLHIV: TPT monitoring can be aligned with ART; Consider drug–drug interactions for CLHIV on ART and TPT ***	If fixed-dose combination (FDC) available, preferred for HIV-negative children < 25 kg	Children of all ages; child-friendly FDC (HR 50 mg/75 mg) is available; If FDC not available, for HIV-negative children <25 kg, WHO recommends 6H for those < 2 years, and 3HP or 6H for those ≥ 2 years of age. Pyridoxine for select patients. ^†^
3HP ^‡^	Isoniazid (H) Rifapentine (P)	Weekly (12 doses)	3		Preferred for PLHIV (>15 years), on TDF, EFV, DTG or RAL-based ART	Not for use in children < 2 years (Lack of data on appropriate rifapentine dosing); Pharmacokinetic and safety studies on 3HP and other rifapentine-based TPT in CLHIV on ART (lopinavir/ritonavir (LPV/r), DTG, NVP) are ongoing; WHO does not recommend 3HP for CLHIV. Take with food containing fat if possible. Pyridoxine for select patients. ^†^
4R	Rifampicin (R)	Daily	4			Children of all ages; No pediatric formulation available: rifampin tablet (300 mg) can be used in older children and adolescents; for younger children, pill crushing or compounding may be necessary.
6H or 9H	Isoniazid (H)	Daily	6 or 9		Preferred for CLHIV on ART	Children of all ages; Preferably use dispersible tablets in children; 3HR may be considered as an alternative regimen for children on EFV-based ART; Monitor for signs of H-induced hepatotoxicity. Pyridoxine for select patients. ^†^
1HP	Isoniazid (H) Rifapentine (P)	Daily (28 doses)	1			1HP is recommended by WHO as an alternative TPT regimen for HIV-negative children aged ≥13 years and > 25 kg and CLHIV ≥13 years if on TDF, EFV, DTG or RAL-based ART. Take with food containing fat if possible. Pyridoxine for select patients. ^†^
Non-Severe Drug-Susceptible TB Disease						
2 months HRZ(E), 2 months HR	Isoniazid (H) Rifampicin (R) Pyrazinamide (Z) ±Ethambutol (E)	Daily	4	Evaluate bi-weekly during intensive phase and monthly thereafter	Children (3 months < 16 years) with non-severe pulmonary or peripheral lymph node disease	Follow definitions for non-severe disease to determine eligibility. For young children, child-friendly, dispersible, and appropriate FDC available (R75/H50/Z150; R75/H50; E100)
Drug-Susceptible TB Disease						
2 months HRZ(E), 4 months HR	Isoniazid(H) Rifampicin (R) Pyrazinamide (Z) ±Ethambutol (E)	Daily	6	Evaluate bi-weekly during intensive phase; monthly thereafter	Children with severe disease and no presumptive drug resistance; children not responding to shorter regimen	Child-friendly formulations available (see above)
2 months HPZM, 2 months (9 weeks) HPM	Isoniazid (H) Rifapentine (P) Pyrazinamide (Z) Moxifloxacin (M)	Daily	4	Evaluate bi-weekly during intensive phase and monthly thereafter	Children ≥ 12 years weighing ≥ 40 kg with drug-susceptible pulmonary TB	Child friendly formulations available for all medications (see above) including moxifloxacin (100 mg). No FDC for full regimen. Cost may be prohibitive in some settings [14].
Drug-Susceptible TB Meningitis or Osteoarticular TB						
2 months HRZE 10 months HR	Isoniazid (H) Rifampicin (R) Pyrazinamide (Z) Ethambutol (E)	Daily	12	Evaluate bi-weekly or at clinician discretion	Children with TBM or osteoarticular TB	The optimal rifampicin dosages are still being evaluated but higher CSF penetration can be achieved with higher dosages (20–30 mg/kg) [14]. Child-friendly formulations available (see above)
6 months HRZEto	Isoniazid (H) Rifampin (R) Pyrazinamide (Z) Ethionomide (Eto)	Daily	6	Evaluate bi-weekly or at clinician discretion	Children with TBM	See above regarding optimal rifampicin dosages. Child-friendly formulations available for RHZE (see above), plus Eto 125 mg

Table adapted from: Nolt D, et al. [38]; and Yuen CM, et al. [76], and informed by the WHO operational handbook [39]; * For more detailed dosing information, including dosing based on weight bands please see WHO operational handbook. ** Monitoring should focus on the following: (1) weight checks; (2) presence of TB symptoms; (3) presence of adverse events related to TBI and TB drug regimen; and (4) adherence to treatment. Routine laboratory monitoring is not necessary for healthy children and adolescents, but should be considered for those with immunocompromise, existing hepatic disease, or on other hepatotoxic medications. *** For more detailed information on TPT regimens to use with ART, please see WHO operational handbook on tuberculosis. ^†^ Select patients include exclusively breastfed infants, children and adolescents on meat or milk-deficient diets, symptomatic CLHIV, children with nutritional deficiencies, and pregnant females. ^‡^ WHO Guidelines are expected out in July 2024 that will directly impact age limitations for 3HR and 3HP.

**Table 3:** Considerations for WHO-Recommended Rapid Diagnostic (WRD) Tests for Pediatric TB Detection *.

Test	Recommended Population	Test Setting	Detects Drug Resistance	Turnaround Time	Recommended Specimen Type ^#^	Sensitivity ^†^	Specificity ^†^
Xpert MTB/RIF	All pediatric subgroups and comorbidities	Near point-of-care (POC) Requires uninterrupted power supply	Yes	<3 h	Sputum	64.6% (55.3 to 72.9) [82]	99.0% (98.1 to 99.5)
					Gastric fluid	73.0% (52.9 to 86.7) [82]	98.1% (95.5 to 99.2)
					NPA	45.7% (27.6 to 65.1) [82]	99.6% (98.9 to 99.8)
					Stool	61.5% (44.1 to 76.4) [82]	98.5% (97.0 to 99.2)
					CSF	54.0% (27.8 to 78.2) [82]	93.8% (84.5 to 97.6)
					Lymph node biopsy	90.4% (55.7 to 98.6) [82]	89.8% (71.5 to 96.8)
					Pleural fluid	25.0% (4.60 to 70.0) [2]	86.4% (66.7 to 95.3)
					Synovial fluid ^‡^	100% [83]	100%
					Urine	66.7% (13 to 100) [84]	100%
Xpert MTB/RIF Ultra	All pediatric subgroups and comorbidities	Near POC Requires uninterrupted power supply	Yes	<3 h	Sputum	72.8% (64.7 to 79.6)	97.5% (95.8 to 98.5)
					Gastric fluid	52.5% (43.9 to 60.9) [82]	100% (87.4 to100.0)
					NPA	45.7% (28.9 to 63.3) [82]	97.5% (93.7 to 99.3)
					Stool	88.9% (51.7 to 98.0) [85]	88.1% (84.7 to 90.8)
						60.3% (51.7 to 68.3) [86]	97.1% (82.9 to 99.8)
Truenat MTB	All pediatric subgroups and comorbidities	Near POC Battery-operated (~8 h)	Yes	<3 h	Sputum	59.4% (48.9 to 69.3) [87]	89.5% (86.6 to 92)
TB-LAMP	All pediatric subgroups and comorbidities	Near POC Requires power supply Other equipment (heating block)	No	~60 min	Sputum	76.5% (50.1 to 93.2) [88]	100% (94.3 to 100)
						84% (63.9 to 95.5) [89]	96% (91.5 to 98.5)
						85.71% (42.13 to 99.64) [90]	100% (95.55 to 100)
						89.8% (79.7 to 96.2) [91]	60.0% (45.9 to 70.0)
LF-LAM (Determine TB LF-LAM Ag Test)	Only HIV positive population with AHD or according to local guidelines	POC No power supply requirement	No	<45 min	Urine	46.59% (32.98 to 60.19) [92]	76.45% (57.07 to 95.82)

* Adapted from WHO operational handbook on tuberculosis. Module 5: management of tuberculosis in children and adolescents [39]. ^#^ No study found evaluating peritoneal fluid, pericardial fluid or blood using Xpert MTB/RIF; CSF or lymph node biopsy using Xpert Ultra; or sputum specimens using Truenat MTB Plus for diagnosing TB in the pediatric population. ^†^ All sensitivity and specificity against culture unless otherwise stated. ^‡^ Compared against composite reference standard using one case study.

**Table 4:** Common challenges, misperceptions, and available solutions for managing TB in children and adolescents along the TB clinical cascade of care.

Important Steps	Common Challenges	Common Misperceptions	Available Solutions
**Screen**			
Appropriately identifying a child with TB as a presumptive TB case	Lack of awareness among healthcare workers about clinical presentation of pediatric TB	The duration of symptoms of TB in children is always chronic	Routinely consider and screen for TB in all sick children and adolescents
	Lack of screening for TB among sick children	Only sick children with a known TB contact should be considered as presumptive TB case	Raise awareness that most children do not have a known TB contact; Older children are commonly exposed outside of the home
**Diagnose**			
Weight	Not determining accurate weight and/or not comparing to previously recorded weights to assess trend	Recent weight loss or poor weight gain are always accurately reported	Emphasize the importance of using established tools to record and compare to previous weights for children under five
Clinical evaluation of a child with presumptive TB	Lack of confidence among HCWs to perform clinical evaluation for TB	The diagnosis of TB in children is always difficult	Provide job aids and outline diagnostic approaches and treatment decision algorithms
Triage	Children with TB can present with “danger signs”	TB is not a cause of acute, severe illness	Follow standard approaches to the management of the sick child, including acute resuscitation and inpatient care when indicated
Evaluate for risk	Nutritional and HIV status not specifically evaluated		Routinely test for HIV and assess nutritional status
Bacteriological confirmation	Lack of sputum sample provided by coughing/spontaneous expectoration	Lack of spontaneously expectorated sputum means laboratory testing cannot be conducted	Range of alternative samples (e.g., stool, urine, nasopharyngeal aspirate) can be tested to confirm TB in children; See Table 1 for SOPs and aids
Clinical diagnosis	Low confidence in pediatric TB diagnosis at peripheral care level; Lack of imaging or ability to interpret imaging, especially CXR, in children	Children with presumptive TB always need referral to specialist; Impossible to determine or distinguish child TB-related abnormalities on CXR	There are available trainings, job aids, clinical decision algorithms, online courses, and other resources to increase awareness and confidence in making a diagnosis and interpreting CXR (Table 1)
**Treat**			
Treatment initiation	Make a decision to treat	Drugs used to treat TB are frequently associated with severe adverse reactions	Training and treatment decision approaches are available along with dosing guidelines (global and national)
Treatment completion	Adherence for full treatment course	Once symptoms have resolved, treatment can be stopped	Patient education and counselling to explain completion is critical; Age-appropriate support which addresses complex needs of school-age children and young adults
**Contact Investigation**			
Coverage of contact investigation	Contact investigation is not routinely initiated or approach is passive (facility-based) only	Contact investigation can only be performed at health facility	Establish contact investigation and management teams that includes community HCW, lay counselors, and peer educators
Active case finding	Focus on young child contacts only for case detection	Contact investigation is only for providing TPT to young child contacts	Raise awareness that contact investigations are a high-yield contributor to case detection for children and adolescents; Establish clear contact management guidelines
**Prevent: TB Preventive Treatment**			
Eligibility for TPT	Uncertainty about which contacts are eligible for TPT	Very difficult to “rule out” active TB	Eligibility can be determined in high-risk exposed children based on symptoms alone
Initiation of TPT	Lack of understanding of rationale for providing medication to a well-appearing child	TPT drugs, such as isoniazid, have many adverse side effects in children	TPT drugs are safe and significantly reduce risk of TB disease when used in children at high risk of developing TB
Completion of TPT	Adherence is challenging for long regimens requiring daily medication		Shorter and weekly treatment options
TPT for MDR/RR TB contacts	Lack of specific TPT recommendation	MDR TB is less infectious than drug-susceptible TB	New guidance is forthcoming from WHO (See TPT: Rapid Communication)

## Data Availability

Not applicable.

## Key Abbreviations and Acronyms

AHD	Advanced HIV Disease
BCG	Bacille Calmette–Guérin Vaccine
CAD	Computer-aided Detection
CFS	Child-friendly Specimen
CLHIV	Children Living with HIV
CRP	C-reactive Protein
CSF	Cerebrospinal Fluid
CT	Computed Tomography
CXR	Chest X-Ray
DS TB	Drug-susceptible TB
DR TB	Drug-resistant TB
EP TB	Extrapulmonary TB
FDC	Fixed-dose Combination
GA	Gastric Aspirate
HCW	Health Care Worker
IGRA	Interferon Gamma Release Assay
IPC	Infection Prevention and Control
LF-LAM	Lateral Flow Urine Lipoarabinomannan Assay
MRI	Magnetic Resonance Imaging
mWRD	Molecular WHO-recommended Rapid Diagnostic Tests
NPA	Nasopharyngeal Aspirate
NTM	Non-tuberculous Mycobacteria
PEPFAR	President’s Emergency Plan for AIDS Relief
PET	Positron Emission Tomography
PLHIV	People Living with HIV
POC	Point-of-Care
PTB	Pulmonary TB
RR TB	Rifampicin-resistant TB
SAM	Severe Acute Malnutrition
SOS	Simple One-Step Method (stool processing)
TBI	TB Infection
TB-LAMP	TB Loop-mediated Amplification
TBM	TB Meningitis
TPT	TB Preventive Treatment
TST	Tuberculin Skin Test
US	Ultrasound
XDR TB	Extensively Drug-resistant TB
Xpert	Xpert MTB/RIF cartridge
Xpert Ultra	Xpert MTB/RIF Ultra cartridge
Medications and Regimens	
ART	Antiretroviral Therapy
E	Ethambutol
Eto	Ethionamide
H	Isoniazid
M	Moxifloxacin
P	Rifapentine
R	Rifampicin
Z	Pyrazinamide
1HP	Prevention regimen: 1 month daily isoniazid and rifapentine
3HP	Prevention regimen: 3 months weekly isoniazid and rifapentine
3HR	Prevention regimen: 3 months daily isoniazid and rifampicin
4R	Prevention regimen: 4 months daily rifampicin
